# Perception of Barriers to Sports Practice in Persons with Disabilities in Chile: A Socio-Ecological Approach

**DOI:** 10.3390/healthcare14182989

**Published:** 2026-09-12

**Authors:** Fernando Muñoz-Hinrichsen, Diana Camargo Rojas, Luís Torres Paz, Marco Kokaly Farah, Noemi Ortega Díaz, Juan López Jofré, Sophie Pombet Ortiz, Martín Lanas Araos, Jorge Pérez-Contreras, Alan Martínez Aros, Felipe Herrera Miranda

**Affiliations:** 1 Laboratorio de Actividad Física, Salud y Rendimiento Humano, Departamento de Kinesiología, Universidad Metropolitana de Ciencias de la Educación, Santiago 7760197, Chile; noemi.ortega2019@umce.cl (N.O.D.); juan.lopez2019@umce.cl (J.L.J.); 2 Centro de Desarrollo de la Investigación (CEDI), Universidad Metropolitana de Ciencias de la Educación, Santiago 7760197, Chile; 3 Departamento del Movimiento Corporal Humano, Universidad Nacional de Colombia, Bogotá 110911, Colombia; dacamargor@unal.edu.co; 4 Universidad Nacional Pedro Ruiz Gallo, Lambayeque 14000, Peru; lutopa1@gmail.com; 5 Escuela de Kinesiología, Universidad de los Andes, Santiago 7550000, Chile; mkokaly@miuandes.cl; 6 Escuela de Ciencias del Deporte y Actividad Física, Facultad de Salud, Universidad Santo Tomás, Santiago 8370003, Chile; s.pombet@alumnos.santotomas.cl (S.P.O.); m.lanas2@alumnos.santotomas.cl (M.L.A.); jperez51@santotomas.cl (J.P.-C.); 7 Escuela de Kinesiología, Facultad de Rehabilitación, Universidad Andrés Bello, Santiago 8370134, Chile; alankine@gmail.com; 8 Escuela de Kinesiología, Facultad de Odontología y Salud, Universidad Diego Portales, Santiago 8370134, Chile; 9 Carrera de Kinesiología, Facultad de Ciencias de la Vida, Universidad Viña del Mar, Viña del Mar 9460000, Chile; fherrera@uvm.cl

**Keywords:** adapted sport, disability, physical activity, socio-ecological model, Chile, human rights

## Abstract

Background: Participation in physical and sports activities is essential for the overall health of persons with disabilities (PWD). However, these individuals encounter multiple environmental and contextual barriers limiting sports engagement. Objective: This study aimed to analyze the perceived barriers to sports participation among PWD in Chile using a socio-ecological framework, evaluating differences by sex and sports practice status while adjusting for potential demographic confounders. Methods: A quantitative, cross-sectional design was conducted with a convenience sample of 245 PWD. Perceptions across four socio-ecological dimensions (intrapersonal, interpersonal, organizational, and community) were measured using the short Spanish version of the Barriers to Physical Activity Questionnaire for People with Mobility Impairments (BPAQ-MI). Non-parametric Mann–Whitney U tests were performed as primary bivariate analyses due to non-normal data distribution, followed by Analysis of Covariance (ANCOVA) models controlling for age and sex. Results: Bivariate analyses showed no significant differences by sex. When comparing sports participants versus non-participants, athletes perceived significantly lower barriers in the organizational (U=5357, p<0.001, r=0.286) and community (U=6216, p=0.020, r=0.172) dimensions. After adjusting for age and sex via ANCOVA, sports practice remained a significant independent predictor of lower organizational barriers ($F=7.814,\;p=0.006,\;\eta_{p}^{2}=0.032$), and a significant interaction between sports practice and sex was identified in the community dimension ($F=4.671,\;p=0.032,\;\eta_{p}^{2}=0.019$). Community-level barriers were rated highest across all groups. Conclusions: Sports participation is associated with a lower perception of organizational barriers, even when controlling for age. The prominent community-level obstacles underscore the need for targeted, context-specific public policies that enhance accessibility and inclusive infrastructure.

## 1. Introduction

Regular physical activity (PA) and sports participation are essential components of physical and psychological health, functioning, and overall quality of life for persons with disabilities (PWD) [1,2,3]. Engagement in structured physical activity supports functional mobility, cardiovascular health, and musculoskeletal strength, while simultaneously mitigating secondary health complications such as cardiovascular disease, metabolic syndrome, and muscle atrophy [3,4,5,6,7,8]. Furthermore, active engagement in physical and sports activities is consistently associated with positive psychological outcomes, including reduced symptoms of anxiety and depression, enhanced self-efficacy, and greater subjective well-being [9,10].

Despite these established benefits, individuals with disabilities globally—and particularly across Latin America—exhibit disproportionately high levels of physical inactivity compared to the general population [4,5]. This participation gap is driven by a complex interplay of personal, physical, attitudinal, and environmental factors [5,6]. In regional contexts such as Chile, structural factors—including limited physical accessibility, inadequate adaptive sport infrastructure, scarce specialized professional training, and financial constraints—significantly restrict opportunities for sustained physical and sports participation among PWD [5,6].

To systematically evaluate these multi-level factors, this study adopts the framework of the International Classification of Functioning, Disability and Health (ICF) [7] integrated with Bronfenbrenner’s socio-ecological model [8] and Hutzler’s adapted physical activity model [9]. Within the ICF framework, contextual environmental factors are conceptualized along a continuum: “barriers” represent external obstacles that restrict social participation and performance, whereas “facilitators” denote environmental or personal resources that enhance functioning and reduce disability [10,11,12]. In the present investigation, environmental factors are evaluated using a standardized barrier-quantification instrument; lower perceived barrier intensity across socio-ecological levels is thus operationally interpreted as representing environments with greater implicit facilitation and lower friction for sports participation [10,11,12].

The socio-ecological approach categorizes these contextual factors into four distinct levels: intrapersonal (individual attitudes, self-efficacy, and impairment characteristics), interpersonal (family, peer, and community social support), organizational (institutional resources, club accessibility, and trained personnel), and community (public infrastructure, built environment, and social attitudes) [13,14,15].

In Chile, legislative and institutional efforts have aimed to promote sports participation among PWD, highlighted by Law 20,978 recognizing adapted and Paralympic sport [16], national physical activity policies [17], and the hosting of major international events such as the Santiago 2023 Parapan American Games.

Given this persistent participation gap, a comprehensive empirical assessment of the environmental and socio-ecological factors influencing physical activity among PWD in Chile is critically needed. Therefore, the primary objective of this study was to evaluate perceived barriers to sports participation among PWD in Chile across four socio-ecological dimensions (intrapersonal, interpersonal, organizational, and community). A secondary objective was to compare barrier perceptions between active sports participants (“athletes”) and non-participants (“non-athletes”), controlling for key demographic confounders such as age and sex.

## 2. Materials and Methods

This study has a quantitative methodology of a comparative and cross-sectional nature, seeking to identify which variables act as facilitators or barriers for people with disabilities in Chile.

### 2.1. Participants

A quantitative, cross-sectional, comparative study was conducted among persons with disabilities residing in Chile. A total sample of 245 adults with disabilities (M_age_ = 41.37 ± 17.58 years; 75.5% male, 24.5% female) participated in the study. Diagnostic distribution across the total sample was as follows: physical impairment (75.4%, *n* = 185), multiple impairments (12.2%, *n* = 30), visual impairment (9.01%, *n* = 22), hearing impairment (2.45%, *n* = 6), and intellectual disability (0.82%, *n* = 2).

Participants were operationalized into two primary comparative groups based on their sports participation status:

Athletes (*n* = 123; 50.2%): Defined as individuals actively engaged in structured, regular sports practice (minimum of 2 sessions per week, ≥120 min total weekly volume) within affiliated adapted sport clubs, sports centers, or Paralympic training programs [4].

Non-Athletes (*n* = 122; 49.8%): Defined as individuals who did not participate in structured sports practice or regular organized physical activity programs [4].

To account for biological sex and sports participation, the sample was further stratified into four sub-cohorts: Male Non-Athletes (*n* = 87; 35.5%), Male Athletes (*n* = 98; 40.0%), Female Non-Athletes (*n* = 35;14.3%), and Female Athletes (*n* = 25; 10.2%). Notable demographic differences were present between sports groups, with athletes being significantly younger (M = 33.28 ± 13.89 years) than non-athletes (M = 49.51 ± 16.94 years; *p* < 0.001), necessitating statistical control for age in subsequent comparative analyses.

A non-probabilistic convenience sampling strategy was employed to recruit participants across central and regional zones of Chile. Recruitment was carried out in direct collaboration with the Chilean Paralympic Committee (COPACHI), regional adapted sports federations, municipal disability departments, local community rehabilitation centers (CCR), and community-based organizations. Recruitment channels included formal institutional invitations, email communications, and direct telephone contact.

Eligibility criteria required participants to: (a) be at least 18 years of age, (b) possess an officially recognized medical or administrative diagnosis of disability, (c) reside in Chile, and (d) possess the cognitive capacity to understand the study purpose and respond to the questionnaire independently or with minimal standardized assistance.

Data collection was conducted via structured face-to-face or virtual synchronized interviews administered by trained research personnel between March 2023 and January 2024. Prior to administration, all participants received a detailed explanation of the study objectives and provided written informed consent.

A post hoc sensitivity analysis conducted in G*Power (version 3.1.9.7; Heinrich-Heine-Universität Düsseldorf, Düsseldorf, Germany) indicated that the final sample (N=245) provided sufficient statistical power (1−β=0.80, α=0.05) to detect small-to-medium effect sizes (d≥0.36;r≥0.18) in bivariate comparisons.

### 2.2. Ethical Considerations

The study protocol was conducted in accordance with the principles of the Declaration of Helsinki and was formally evaluated and approved by two institutional ethics review boards: the Ethics Committee of Universidad Santo Tomás, Chile (Approval No. 166/2024, covering data collection protocols in sports centers) and the Ethics Committee of Universidad Diego Portales, Chile (Approval No. 12/2022, covering the primary multi-center research framework).

### 2.3. Instrument

The Barriers to Physical Activity Questionnaire for People with Mobility Impairments (BPAQ-MI) in its reduced Spanish version was used [18]. According to the original validation study of this Spanish adaptation [18], the instrument demonstrated excellent internal consistency with an overall Cronbach’s alpha of 0.92, as well as good reliability across its subscales: The intrapersonal (α = 0.740), interpersonal (α = 0.891), organizational (α = 0.893), and community (α = 0.914) factors also demonstrated good reliability. Furthermore, it has a good overall fit: X2371 = 1580.965 (*p* < 0.001); CFI = 0.97; RMSEA = 0.064 (90% CI = 0.061–0.067). In the present study, internal consistency across dimensions was satisfactory: Intrapersonal (α=0.710, ω=0.724), Interpersonal (α=0.60, ω=0.63), Organizational (α=0.67, ω=0.69), and Community (α=0.72, ω=0.74).

It turned out to be a four-dimensional, 29-item questionnaire that maintained a balance among the four socio-ecological levels (intrapersonal level = 7 items; interpersonal level = 7 items; organizational level = 8 items; community level = 7 items). The new instrument was named the Barriers to Physical Activity Questionnaire for People with Disabilities. To interpret the questionnaire, a higher score (4 being the maximum) indicates greater barriers to participation, while a lower score (0 being the minimum) indicates a greater number of facilitators.

### 2.4. Data Analysis

Descriptive statistics are presented as frequencies (n) and percentages (%) for categorical variables, and as means (M), standard deviations (SD), medians (Mdn), and interquartile ranges (IQR) for continuous variables.

Normality of distribution for the four dependent variables—the Intrapersonal, Interpersonal, Organizational, and Community subscales of the BPAQ-MI—was evaluated using the Shapiro–Wilk test alongside skewness and kurtosis metrics. Because the subscales significantly deviated from normality across multiple subgroups (p<0.05) and homoscedasticity was violated in specific comparisons (Levene’s test p<0.05), non-parametric Mann–Whitney U tests were established as the primary bivariate statistical method to evaluate group differences (Sex: Male vs. Female; Sports Status: Athletes vs. Non-Athletes). For all bivariate comparisons, rank-biserial correlation (r) was calculated as the effect size metric, with values of 0.10, 0.30, and 0.50 representing small, medium, and large effect sizes, respectively. To control for inflated Type I error due to multiple subgroup testing, p-values were adjusted using the Benjamini–Hochberg False Discovery Rate (FDR) procedure.

To address demographic confounding, specifically the age disparity between active athletes and non-athletes, a series of Analyses of Covariance (ANCOVA) were performed for each socio-ecological dimension. Models included Sports Status (Athlete vs. Non-Athlete), Sex (Male vs. Female), and their interaction term (Sports Status×Sex) as fixed factors, with Age entered as a continuous covariate. Partial Eta-squared ($\eta_{p}^{2}$) was reported as the effect size for ANCOVA models, interpreted as small (0.01), medium (0.06), and large (0.14). Statistical significance was set at two-tailed α=0.05 across all tests.

The analysis considered the 0–4 Likert scale, and the dependent variables were the subscales of the BPAQ-MI instrument (intrapersonal, interpersonal, and organizational).

Statistical analyses were performed using Jamovi software (version 2.3) and the R software environment (version 4.1). Missing data were audited before analysis; questionnaires with >5% missing responses were excluded, while isolated missing values (<1%) were handled via full-information máximum-likelihood estimation.

## 3. Results

The total sample comprised 245 participants (185 men, 75.5%; 60 women, 24.5%). Half of the sample actively participated in organized adaptive sports (n=123, 50.2%), while 122 participants (49.8%) were non-athletes. A significant age disparity was observed between groups, with active athletes being substantially younger (M=33.28±13.89 years; Mdn=30.00, IQR=18.00) than non-athletes (M=49.51±16.94 years; Mdn=54.00, IQR=27.00).

Descriptive metrics for the four socio-ecological subscales of the BPAQ-MI across sub-cohorts (stratified by sex and sports participation) are presented in Table 1.

Bivariate comparisons evaluating perceived barrier intensity across biological sex (Men, n=185 vs. Women, n=60) showed no statistically significant differences in any of the four socio-ecological dimensions. Specifically, in the Intrapersonal dimension, scores reported by male participants (Mdn=1.86, IQR=1.14) were equivalent to those of female participants (Mdn=1.79, IQR=1.32; Mann–Whitney U=5530, p=0.967, r=0.004).

In the Interpersonal dimension, scores for women (Mdn=1.71, IQR=1.47) were slightly higher than those for men (Mdn=1.43, IQR=1.29), though this difference was not statistically significant (U=4970, p=0.223, r=0.105). Similarly, in the Organizational dimension, barrier scores reported by men (Mdn=1.75, IQR=1.00) did not differ significantly from those reported by women (Mdn=1.63, IQR=0.78;U=5126, p=0.374, r=0.076).

Finally, the highest barrier scores across both cohorts were recorded in the Community dimension (Mdn=2.29, IQR=0.85 for men; Mdn=2.29, IQR=0.85 for women), with non-parametric testing confirming no significant sex-based disparity (U=5298, p=0.596, r=0.046). Bivariate sex comparisons are summarized in Table 2.

### 3.1. Bivariate Comparisons by Sports Participation Status

Non-parametric bivariate analyses comparing active sports participants (“Athletes”, n=123) versus non-participants (“Non-Athletes”, n=122) demonstrated significant differences across multiple socio-ecological levels.

In the Intrapersonal dimension, non-athletes reported descriptive barrier scores (Mdn=2.00, IQR=1.39) slightly higher than athletes (Mdn=1.71, IQR=1.00), though this difference was not statistically significant (U=6684, p=0.140, r=0.109). Conversely, the Interpersonal dimension showed significantly higher perceived barrier intensity among non-athletes (Mdn=1.71, IQR=1.43) compared to active athletes (Mdn=1.29, IQR=1.15;U=6402, p=0.047, r=0.147). The largest bivariate contrast was identified within the Organizational dimension. Non-athletes perceived substantially greater organizational obstacles (Mdn=1.88, IQR=1.00) than athletes (Mdn=1.50, IQR=0.63), demonstrating a highly significant statistical difference (U=5357, p<0.001, r=0.286).

Finally, in the Community dimension, non-athletes perceived significantly greater environmental and architectural barriers (Mdn=2.43, IQR=0.97) compared to active athletes (Mdn=2.29, IQR=0.85; U=6216, p=0.020, r=0.172). Due to non-normal distribution and heteroscedasticity across subgroups (Shapiro–Wilk p<0.05; Levene’s test p=0.001), the non-parametric Mann–Whitney *U* test was confirmed as the appropriate primary bivariate decision metric. Bivariate comparisons by sports participation status are detailed in Table 3.

### 3.2. Multivariable ANCOVA Models Controlling for Demographic Confounders

To evaluate whether observed differences in perceived barriers were independently driven by sports participation or confounded by age and sex disparities, a series of Analysis of Covariance (ANCOVA) models were conducted.

In the Organizational dimension, the overall model was statistically significant ($F\left(4,\;240\right)=4.396,\;p=0.002$). After controlling for Age ($F=0.768,\;p=0.382,\;\eta_{p}^{2}=0.003$) and Sex ($F=1.923,\;p=0.167,\;\eta_{p}^{2}=0.008$), Sports Status remained a statistically significant independent predictor of lower perceived organizational barriers ($F=7.814,\;p=0.006,\;\eta_{p}^{2}=0.032$).

In the Community dimension, the overall model was also significant ($F\left(4,\;240\right)=3.128,\;p=0.016$). While the main effect of Sports Status was not independently significant (F=0.019, p=0.892), a statistically significant interaction term between Sports Status × Sex was identified ($F=4.671,\;p=0.032,\;\eta_{p}^{2}=0.019$), indicating that the impact of sports participation on reducing community barrier perceptions is moderated by sex.

Models for the Intrapersonal ($F\left(4,\;240\right)=1.276,\;p=0.280$) and Interpersonal ($F\left(4,\;240\right)=1.233,\;p=0.298$) dimensions were not statistically significant after covariate adjustment.

## 4. Discussion

The objective of this study was to analyze perceived barriers and implicit facilitators affecting physical activity and sports participation among persons with disabilities in Chile, utilizing a socio-ecological framework as a theoretical foundation [12,13]. By examining multi-level environmental and personal factors across intrapersonal, interpersonal, organizational, and community dimensions, and controlling for demographic confounders such as age and sex, this investigation provides empirical insights into the contextual friction shaping adaptive sports engagement in Chile.

One of the central pillars of this discussion is the urgent need to generate more effective and adapted public policies, built not only from a general normative perspective, but also from empirical knowledge of the real and everyday barriers faced by persons with disabilities. Inclusive policies must be built upon accurate diagnoses, situated territorially and culturally, that identify both existing obstacles and opportunities. The International Classification of Functioning, Disability and Health (ICF) model provides a key basis for this transformation, considering that functioning and disability are the result of the interaction between health conditions and contextual factors [12]. As the literature confirms, lack of access to resources, adequate spaces, institutional support, and social networks can act as limiting barriers [19]. Therefore, transformative policy must look beyond the individual and recognize the decisive influence of the physical, organizational, and social environment.

In this vein, it is pertinent to incorporate the perspective of functional diversity across all social policies, moving beyond a welfare-based approach and promoting holistic human development [20]. Universal design, accessibility, and the direct participation of persons with disabilities themselves in policy formulation are key principles for inclusive and effective governance [21]. Furthermore, as DePauw and Gavron [22] argue, active participation in sports is not only a right but also a tool for empowerment and building citizenship. This implies that policies must guarantee equitable access and participation, while simultaneously fostering cultural changes in the communities, institutions, and social actors that act as mediators of these opportunities.

Regarding sports participation, one of the most relevant empirical findings of this study is that persons with disabilities engaged in structured sports practice perceive lower barrier intensity compared to non-athletes. However, in contrast to preliminary descriptive observations, statistical support for this association is dimension-specific when evaluated through multivariable modeling. When controlling for age and sex through ANCOVA, sports participation remained significantly associated with lower perceived barriers exclusively within the organizational dimension ($F=7.814,\;p=0.006,\;\eta_{p}^{2}=0.032$), while a significant interaction between sports status and sex emerged in the community dimension ($F=4.671,\;p=0.032,\;\eta_{p}^{2}=0.019$). In contrast, differences observed in the interpersonal dimension were statistically significant only in unadjusted bivariate testing (U=6402, p=0.047, r=0.147), and the intrapersonal dimension exhibited no statistically significant divergence between athletes and non-athletes (U=6684, p=0.140, r=0.109). Therefore, findings regarding intrapersonal factors must be interpreted strictly as non-significant descriptive trends rather than statistically supported effects.

From a health and psychosocial perspective, physical activity and sports engagement are consistently associated with overall well-being and functional benefits for persons with disabilities [9]. Regular participation in physical activity is linked to reduced depressive symptoms, improved self-esteem, better sleep quality, and higher self-efficacy, which may support greater agency and social participation [23]. At the organizational level, adapted sports clubs, fitness centers, and specialized programs function as key structural facilitators when equipped with accessible facilities, adapted gear, and trained personnel [24]. The markedly lower organizational barriers reported by active athletes (Mdn=1.50 vs. 1.88 in non-athletes) indicate that structured sports networks provide direct access to institutional resources that attenuate environmental friction. Nevertheless, as observed across Latin American contexts, many of these adapted sports opportunities remain geographically centralized in urban hubs, leaving rural and unaffiliated populations underserved. This highlights the critical need to expand inclusive sports networks, decentralize offerings, and enhance specialized workforce capacity in adapted physical activity [24].

At the intrapersonal level, although group differences did not reach statistical significance, individual factors such as self-motivation, fear of injury, or perceived functional capacity remain relevant elements within Hutzler’s adapted physical activity framework [7]. While active engagement correlates with enhanced personal confidence, individual attitudes interact continuously with surrounding social and physical environments [7,12]. Overall, sports participation is positively associated with lower institutional friction, providing a valuable avenue for promoting health, subjectivities, and active citizenship among persons with disabilities [22].

### Limitations and External Validity

Several methodological limitations of this study must be acknowledged. First, the cross-sectional design precludes establishing causal inferences or directionality. It remains plausible that reverse causality is present: individuals who naturally encounter or perceive fewer environmental and organizational barriers may be more inclined to initiate and sustain sports practice, rather than sports participation directly reducing barrier perception. Prospective longitudinal or intervention designs are required to evaluate potential protective or transformative mechanisms over time.

Second, the study relied on a non-probabilistic convenience sampling strategy, which introduces potential selection bias and limits external validity. The sample was heavily skewed toward male participants (75.5%) and individuals with physical impairments (75.4%), with minimal representation of intellectual (0.82%) or sensory (11.46%) disabilities. Consequently, findings are primarily generalizable to urban-dwelling adult males with physical disabilities affiliated with sports or community networks and should be generalized to other disability populations or rural settings with caution.

Third, although the short Spanish BPAQ-MI demonstrated acceptable sample-specific reliability (α/ω=0.60−0.74), the instrument was originally validated specifically for people with mobility impairments [18]. Measurement invariance across different disability types was not formally evaluated in this study, which represents a psychometric limitation when applying mobility-focused instruments across heterogeneous disability subgroups 18.

Finally, despite applying multivariable ANCOVA modeling to adjust for the observed age gap between active athletes (M=33.28 years) and non-athletes (M=49.51 years), residual confounding by unmeasured sociodemographic variables (e.g., socioeconomic status, income, educational level, or time since disability onset) cannot be entirely excluded. Future studies employing stratified probability sampling, longitudinal tracking, and psychometric invariance testing across diagnostic subgroups are recommended.

## 5. Conclusions

This study provides empirical evidence on the multi-level environmental barriers associated with physical activity and sports participation among persons with disabilities in Chile. Through a socio-ecological framework, three primary conclusions and actionable implications emerge:

1. Differentiated Association by Socio-Ecological Dimension: Structured sports participation is significantly associated with a lower perception of organizational barriers ($p=0.006,\;\eta_{p}^{2}=0.032$), an effect that remains robust after adjusting for age and sex. Bivariate associations were also observed in the interpersonal and community dimensions, whereas intrapersonal barrier perceptions showed no statistically significant divergence between athletes and non-athletes. Overall effect sizes were small-to-moderate, indicating that while sports participation connects individuals to organizational resources, environmental friction persists across macro-systemic levels.
2. Priority Target for Policy Intervention (Community Level): The community dimension consistently recorded the highest barrier scores across all sub-cohorts (Mdn=2.29−2.43). This underscores that public infrastructure, accessible urban transport, and negative social attitudes represent the primary bottlenecks restricting active lifestyles for persons with disabilities in Chile. Furthermore, the significant interaction between sports status and sex (p=0.032) highlights the need for gender-sensitive community adaptations.
3. Prioritized Recommendations for Policy and Practice:
  ○ Community-Level Interventions (High Priority): Public investment should prioritize universal accessibility in municipal sports facilities, public parks, and urban transit systems, alongside community awareness campaigns to eliminate attitudinal barriers.
  ○ Organizational-Level Capacity Building: Decentralize adapted sports programs outside urban hubs, increase public funding for local sports clubs, and mandate specialized training in adapted physical activity for sports administrators and physical education professionals.
  ○ Interpersonal and Intrapersonal Support: Establish community-based peer support networks and inclusive family-oriented programs to facilitate the transition of inactive individuals into sports networks.

### Future Research Directions

Future investigations should employ prospective longitudinal designs to establish causality and evaluate intervention efficacy over time. Furthermore, studies utilizing probability sampling, evaluating psychometric measurement invariance across diagnostic subgroups (physical, sensory, and intellectual), and accounting for socioeconomic status are warranted to enhance generalizability across Latin America.

## Figures and Tables

**Table 1:** Description of participants considering biological sex, sports practice and dimensions of the BPAQ-MI.

								Shapiro–Wilk	
Dimension	Sex	Sports Practice	*n*	M	Mdn	*SD*	*IQR*	*W*	*p*
Intrapersonal	Men	No	87	2.04	2.00	0.960	1.350	0.978	0.150
		Yes	98	1.79	1.64	0.829	1.150	0.966	0.011 *
	Women	No	35	1.88	2.00	0.993	1.640	0.932	0.032 *
		Yes	25	1.94	1.71	0.815	1.000	0.958	0.372
Interpersonal	Men	No	87	1.56	1.57	0.938	1.280	0.978	0.142
		Yes	98	1.33	1.29	0.797	1.113	0.970	0.025 *
	Women	No	35	1.63	1.71	0.919	1.575	0.953	0.139
		Yes	25	1.47	1.43	0.806	1.280	0.966	0.555
Organizational	Men	No	87	2.06	2.00	0.832	1.130	0.987	0.562
		Yes	98	1.66	1.50	0.596	0.630	0.933	<0.001 **
	Women	No	35	1.79	1.75	0.764	0.940	0.972	0.506
		Yes	25	1.62	1.50	0.525	0.630	0.930	0.087
Community	Men	No	87	2.43	2.43	0.756	1.000	0.970	0.038
		Yes	98	2.15	2.21	0.590	1.000	0.975	0.056
	Women	No	35	2.16	2.14	0.745	1.290	0.961	0.249
		Yes	25	2.32	2.29	0.414	0.570	0.916	0.042 *

Note: n = sample size per sub-cohort; SD = Standard Deviation; IQR = Interquartile Range ($Q_{3}-Q_{1}$); W = Shapiro–Wilk test statistic. A p-value <0.05 indicates a significant departure from normal distribution (* p<0.05, ** p<0.001). Higher score values (scale 0–4) indicate greater perceived barriers.

**Table 2:** Non-parametric bivariate comparisons of perceived barriers across biological sex (N=245).

Dimension	Group	*n*	M	Mdn	*SD*	*IQR*	*p*	Adjusted *p* (FDR)	Effect Size
Intrapersonal	Men	185	1.91	1.86	0.90	1.14	0.96	0.96	r = 0.003
	Women	60	1.90	1.79	0.91	1.31			
Interpersonal	Men	185	1.44	1.43	0.87	1.29	0.22	0.44	r = 0.104
	Women	60	1.56	1.71	0.87	1.46			
Organizational	Men	185	1.85	1.75	0.74	1.00	0.37	0.49	r = 0.076
	Women	60	1.72	1.63	0.67	0.78			
Community	Men	185	2.28	2.29	0.68	0.85	0.59	0.59	r = 0.045
	Women	60	2.23	2.29	0.62	0.85			

Note: n = sample size; SD = Standard Deviation; IQR = Interquartile Range ($Q_{3}-Q_{1}$). Mann–Whitney U test applied as the primary non-parametric method due to non-normal distribution (p<0.05 in Shapiro–Wilk test). Rank-biserial correlation (r) reported as effect size (0.10= small, 0.30= medium, 0.50= large). FDR p-values adjusted using the Benjamini–Hochberg procedure.

**Table 3:** Non-parametric bivariate comparisons of perceived barriers by sports participation status (N=245).

Dimension	Group	*n*	M	Mdn	*SD*	*IQR*	*p*	Adjusted *p* (FDR)	Effect Size
Intrapersonal	No	122	1.99	2.00	0.96	1.39	0.14	0.18	r = 0.109
	Si	123	1.82	1.71	0.82	1.00			
Interpersonal	No	122	1.58	1.71	0.92	1.43	0.04 *	0.09	r = 0.147
	Si	123	1.36	1.29	0.79	1.15			
Organizational	No	122	1.98	1.88	0.81	1.00	<0.001 **	<0.001 **	r = 0.286
	Si	123	1.65	1.50	0.58	0.63			
Community	No	122	2.35	2.43	0.76	0.96	0.02 *	0.08	r = 0.172
	Si	123	2.19	2.29	0.56	0.85			

Note: n = sample size; SD = Standard Deviation; IQR = Interquartile Range ($Q_{3}-Q_{1}$). Mann–Whitney U test applied as the primary non-parametric method due to non-normal data distribution. Rank-biserial correlation (r) reported as effect size. FDR p-values adjusted using the Benjamini–Hochberg procedure (* p<0.05, ** p<0.001).

## Data Availability

The data presented in this study are available on request from the corresponding author: F.M.-H.: Fernando Muñoz-Hinrichsen; D.C.R.: Diana Camargo Rojas; L.T.P.: Luís Torres Paz; M.K.F.: Marco Kokaly Farah; N.O.D.: Noemi Ortega Díaz; J.L.J.: Juan López Jofré; S.P.O.: Sophie Pombet Ortiz; M.L.A.: Martín Lanas Araos; J.P.-C.: Jorge Pérez-Contreras; A.M.A.: Alan Martinez Aros; F.H.M.: Felipe Herrera Miranda.

## Abbreviations

The following abbreviations are used in this manuscript:

PWD	People with disabilities
ICF	International Classification of Functioning, Disability and Health
BPAQ-MI	The Barriers to Physical Activity Questionnaire for People with Mobility Impairments
